# Six years of wild bee monitoring shows changes in biodiversity within and across years and declines in abundance

**DOI:** 10.1002/ece3.9190

**Published:** 2022-08-12

**Authors:** Nash E. Turley, David J. Biddinger, Neelendra K. Joshi, Margarita M. López‐Uribe

**Affiliations:** ^1^ Department of Entomology, Center for Pollinator Research The Pennsylvania State University Pennsylvania USA; ^2^ Intercollege Graduate Degree Program in Ecology The Pennsylvania State University Pennsylvania USA; ^3^ Department of Entomology and Plant Pathology University of Arkansas Fayetteville Arkansas USA

**Keywords:** community phylogenetics, insect declines, monitoring, phenology, pollinators, wild bees

## Abstract

Wild bees form diverse communities that pollinate plants in both native and agricultural ecosystems making them both ecologically and economically important. The growing evidence of bee declines has sparked increased interest in monitoring bee community and population dynamics using standardized methods. Here, we studied the dynamics of bee biodiversity within and across years by monitoring wild bees adjacent to four apple orchard locations in Southern Pennsylvania, USA. We collected bees using passive Blue Vane traps continuously from April to October for 6 years (2014–2019) amassing over 26,000 bees representing 144 species. We quantified total abundance, richness, diversity, composition, and phylogenetic structure. There were large seasonal changes in all measures of biodiversity with month explaining an average of 72% of the variation in our models. Changes over time were less dramatic with years explaining an average of 44% of the variation in biodiversity metrics. We found declines in all measures of biodiversity especially in the last 3 years, though additional years of sampling are needed to say if changes over time are part of a larger trend. Analyses of population dynamics over time for the 40 most abundant species indicate that about one third of species showed at least some evidence for declines in abundance. Bee family explained variation in species‐level seasonal patterns but we found no consistent family‐level patterns in declines, though bumble bees and sweat bees were groups that declined the most. Overall, our results show that season‐wide standardized sampling across multiple years can reveal nuanced patterns in bee biodiversity, phenological patterns of bees, and population trends over time of many co‐occurring species. These datasets could be used to quantify the relative effects that different aspects of environmental change have on bee communities and to help identify species of conservation concern.

## Graphical abstract

Six years of bee monitoring shows that bee biodiversity is highly dynamic from month to month and across years. Looking across 40 species, 1/3 show evidence of declining abundance over time.

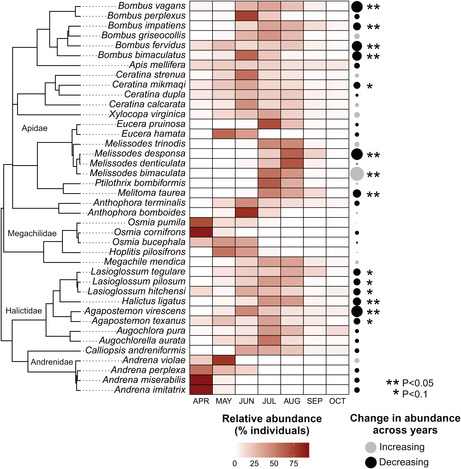

Perplexing bumble bee (*Bombus perplexus*) is one of the species that did not change in abundance over 6 years of our bee monitoring. Also, for a bumble bee, this species had a narrow phenological breadth as they were captured mostly in the month of June. Here, the bee is visiting a purple coneflower (*Echinacea purpurea*) which is one of the plant species sown into the wildflower strip where we monitored bee communities. Photo credit: Nash Turley.
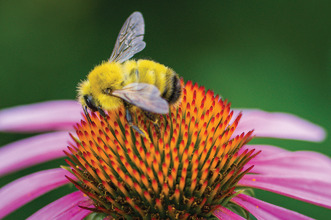

## 1 INTRODUCTION

Pollinators facilitate reproduction for over 80% of flowering plants (Ollerton et al., 2011) and increase the yield, to varying extent, of 75% of crop species (Klein et al., 2007). Bees are one of the most important group of pollinators (Neff & Simpson, 1993) thus detecting changes in bee biodiversity is important for developing pollinator management plans to sustain wild plant communities while maximizing crop yields (Garibaldi et al., 2013; Winfree et al., 2018). A variety of bee monitoring efforts have found troubling declines among wild bees (Biesmeijer et al., 2006; Goulson et al., 2015; Potts et al., 2010). For example, some species have had substantial range contractions and declines in abundance, especially bumble bees in North America and Europe (Bartomeus et al., 2013; Cameron et al., 2011; Williams & Osborne, 2009). Overall predictions show that wild bee abundance is falling in over 23% of the United States' land area (Koh et al., 2016) and the number of bee species observed around the world in museum collections and from community science observations has dropped by 25% from 1990 to 2015 (Zattara & Aizen, 2021). Because of their importance and growing evidence of declines, bee monitoring efforts that build a better understanding of the dynamics of bee biodiversity are important for developing plans that can lead to conserving and restoring wild bee populations (LeBuhn et al., 2013; Winfree, 2010; Woodard et al., 2020).

Bee biodiversity can be measured in a variety of ways, all of which give unique insights into the dynamics of populations and communities within and across years. Biodiversity within populations or communities can be measured in a variety of ways including: abundance, richness, diversity, phylogenetic structure, and community composition. Total individual abundance can provide information on the times within years that are most favorable for most species. Data on the abundance of individual species across years are critical for understanding if species population trends are stable, increasing, or declining over time. For bees, these types of abundance data are often not available because of a lack of repeated and standardized sampling over time (Portman et al., 2020). Richness, or the number of species, is another metric of interest in biodiversity studies, particularly from a restoration and conservation perspective because maintaining or increasing native richness is often an explicit goal in restoration and conservation programs (Tonietto & Larkin, 2018). However, richness can sometimes provide limited unique information because the detection of species is highly dependent on sample sizes, as more individuals counted tend to lead to higher species detection. Diversity metrics help solve these limitations by summarizing aspects of richness and relative abundance among species (evenness) in a single estimator. For example, measures like inverse Simpson's and rarefied richness represent the effective number of species and provide biodiversity measures that are independent of abundance‐driven changes in richness (Jost, 2006). Biodiversity can also be measured in a way that incorporates information about the evolutionary distance that is present among all species in a community using tools from the field of community phylogenetics (Webb et al., 2002). A community with many closely related species is more clustered, while a community populated with distantly related species is more even (also called overdispersed). Finally, community composition uses the presence–absence or relative abundances of all species to determine how similar communities are. The multivariate nature of these measures means it is possible to detect changes among communities even if overall richness, abundance, and diversity are the same. For this reason, community composition measures can be particularly powerful in detecting changes over time or responses of communities to environmental degradation or restoration (Nerlekar & Veldman, 2020).

Adult bee communities are highly dynamic within years making standardized and season‐long sampling necessary to accurately characterize seasonal variations in their biodiversity (Leong et al., 2016). In temperate climates, bees overwinter as larvae, pupae, or adults and then emerge in spring or summer in response to a variety of environmental cues (Cane, 2021). But the time of the year in which bees are active (seasonality) and the duration of their period of activity (phenological breadth) vary greatly among species, resulting in ever‐shifting communities within each year (Kammerer et al., 2021; Ogilvie & Forrest, 2017). Some studies have investigated changes in bee community composition using continuous standardized sampling across the entire period of adult bee activity (e.g., Heithaus, 1979; Joshi et al., 2015; Kammerer et al., 2016; Leong et al., 2016; Neave et al., 2020; Roubik et al., 2021; Stemkovski et al., 2020; Wilson et al., 2008). However, for many bee communities, we still lack a detailed understanding of how community biodiversity and composition change from month to month. Relatively fewer studies have compared the phenological patterns, both seasonality, and phenological breadth, for many co‐occurring species (but see Stemkovski et al., 2020). One reason for this is that the focus of many bee community studies are in agricultural settings where the blooming period of crops is only over a small period of time (e.g., Grab et al., 2019; Graham et al., 2021; Russo et al., 2015).

Bee abundance and richness can also change greatly across years and there are pros and cons to different methods of assessing changes over time (Aldercotte et al., 2022; Graham et al., 2021; Ogilvie et al., 2017). One method for studying changes in bee species over time is to compare historical records with more recent collections (Bartomeus et al., 2013; Burkle et al., 2013; Cameron et al., 2011; Mathiasson & Rehan, 2019; Wood et al., 2019). This approach has the benefits of looking at changes over long time periods and may not require any additional collections of bees from the wild. However, these types of studies are typically only able to investigate changes in a subset of species that are relatively common or popular among collectors (like bumble bees) and are most informative to detect changes in species’ geographic distribution (Cameron et al., 2011; Mathiasson & Rehan, 2019; Wood et al., 2019). While it is possible to estimate changes in species' relative abundance over time from museum collections data (e.g., Bartomeus et al., 2013) these estimates can be skewed by changes in collection methods over time (Gotelli et al., 2021). Furthermore, relative abundances are a community‐level pattern and do not give direct measures of species' population‐level changes over time (Gotelli et al., 2021). An alternative approach is to conduct standardized sampling, often using passive traps, in the same locations across multiple years (Gezon et al., 2015; Graham et al., 2021; Iserbyt & Rasmont, 2012; Martins et al., 2013; Onuferko et al., 2018). These sampling approaches have the benefit of providing more direct measures of changes in population‐level abundances, and potentially for a wide variety of co‐occurring species. However, most standardized sampling does involve collecting a large number of wild bees which can lead to ethical concerns (Gezon et al., 2015; Portman et al., 2020). Also, it can be logistically difficult to continue these types of standardized sampling for a long enough period of time or with enough sample sites to have the statistical power to detect larger population trends (Didham et al., 2020; Lebuhn et al., 2013; Tronstad et al., 2022). Not surprising, standardized longitudinal studies of bee communities are rare but the existing ones have reported high year‐to‐year variation in abundance and a mix of species that are stable, increasing, and decreasing in abundance over time (Aldercotte et al., 2022; Graham et al., 2021; Herrera, 2019; Ogilvie et al., 2017; Onuferko et al., 2018; Roubik et al., 2021). Jointly, these studies have increased the need and interest in formal monitoring of bee biodiversity to assess if there are declines and potential links to decreasing pollination services (Woodard et al., 2020).

In this study, we conducted standardized bee collections across 6 years in Southern Pennsylvania, USA to characterize changes in bee community biodiversity and changes in abundance of specific species. Specifically, we quantified abundance, richness, diversity, phylogenetic structure, and composition of bee communities between months and years. We collected bees continuously from April through October using passive Blue Vane traps. With this, we asked the following specific research questions:

1. How does bee biodiversity change within years?
2. How does bee biodiversity change across years?
3. How do seasonal patterns differ among bee families and species?
4. Do bee families and species differ in their changes in abundance over time?

## 2 METHODS

### 2.1 Study site

**FIGURE 1:**
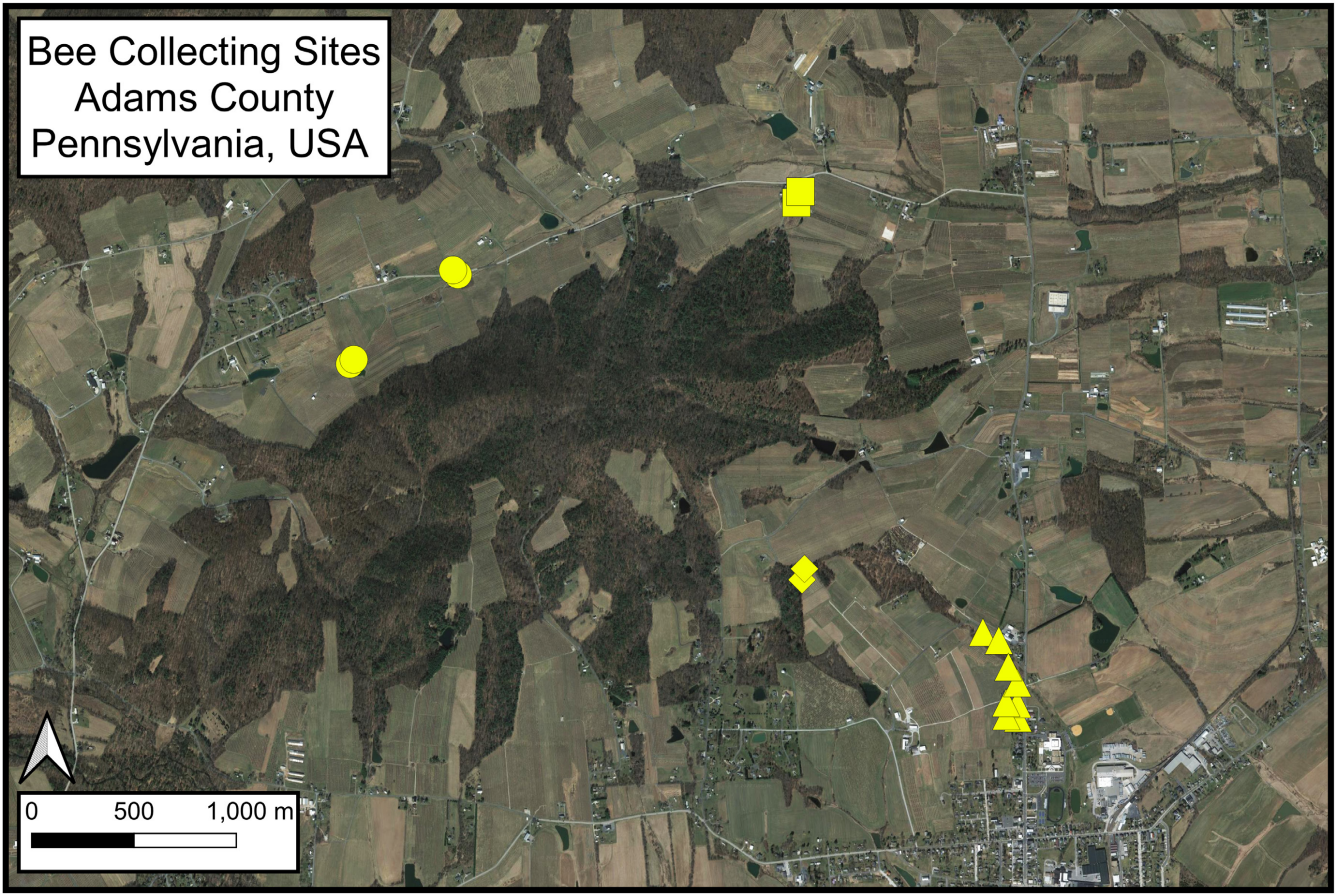
Map of collection sites in Adams County, Pennsylvania, USA. Each yellow marker is the location of a single Blue Vane trap which was left out to capture bees from April to October for 6 years. The four shapes show the collection sites that were closer than 900 m and were lumped together for data analysis. The town of Biglerville is seen at the bottom right.

Our study took place between 2014 and 2019 at the Pennsylvania State Fruit Research and Extension Center in Adams County, Pennsylvania, USA (39.935226, −77.254530) and nearby apple orchards. This site has an average yearly rainfall of 112 cm, average summer temperature ranging from 16 to 28°C, and average winter temperatures of −5 to 5°C (Biddinger et al., 2018). The landscape is hilly with well‐drained soils and the broader area is approximately 56% broadleaf forest fragments, 25% pastureland, 9% developed areas, and 8% commercial orchards (Biddinger et al., 2018). All orchards were managed under growers' choice of conventional pest management programs that use a variety of pesticide classes including: insect growth regulators, anthranilic diamide, tetramic acid, microbials, and neonicotinoid insecticides (Biddinger et al., 2018). We sampled bees at 8 locations adjacent to 4 different active apple orchards. Sampling locations were within 150 m of orchards and 250 m of a forest fragment (Figure 1), which have diverse plant and pollinator communities (Kammerer et al., 2016). Often orchards rely, in part, on managed honey bee colonies for pollination, which have the potential to negatively impact native bee populations (Mallinger et al., 2017). However, our sampling sites did not have managed honey bee hives within 2 km and growers managing the adjacent orchards had not rented honey bees for at least 15 years.

Our bee monitoring traps were located within perennial wildflower strips approximately 50 m × 10 m in size that were sown between 2 and 3 years before the beginning of our study. Wildflower sites used in this study were established and managed using the specific planting guidelines developed by the Pennsylvania USDA‐NRCS and the Xerces Society for Invertebrate Conservation (NRCS, 2011). They were sown with 21 species of native forbs and grasses sourced from a local native seed supplier (Ernst Conservation Seed). All wildflower sites were mowed once a year and received spot sprays of common selective herbicides to control nonnative plants as needed.

### 2.2 Bee collections

We trapped bees continuously from April to October from 2014 to 2019 using Blue Vane traps (BanfieldBio Inc.). A previous study in this region showed that Blue Vane traps collect a higher abundance and total richness of bees than colored bowl traps, also called pan traps (Joshi et al., 2015). Although the overall community composition of bees captured in Blue Vane traps was different from bowl traps, nearly all species were more likely to be captured in Blue Vane traps over bowls, except some *Andrena* and *Lasioglossum* species (Joshi et al., 2015). In our study, Blue Vane traps were filled with about 7 cm of 60% ethylene glycol (Supertech® Wal‐Mart Stores, Inc.) and hung from posts about 1.5 m off the ground. At each of our 8 locations, we placed 2 traps 25 m apart (Figure 1). Traps were left outside continuously from April to October every year and traps were replaced each year in case wear over time decreased their attractiveness. Each week, all specimens were removed and the ethylene glycol was replaced. Bee specimens were separated from other insects collected in the traps and stored in 70% alcohol until they were washed, pinned, and labeled. All bees were identified to the species level except 14 individuals that were removed from analyses because of uncertain species‐level identification. For bee identification, we used published dichotomous keys (Michener, 2000; Michener et al., 1994; Mitchell, 1960, 1962) and various interactive bee identification guides available at Discover Life (http://www.discoverlife.org). Species identifications were conducted by David Biddinger (Pennsylvania State University), Robert Jean (Senior Entomologist, Environmental Solutions and Innovations, Inc.), Jason Gibbs (University of Manitoba), and Sam Droege (United States Geological Survey). All specimens from this study are stored at the Pennsylvania State Fruit Research and Extension Center, Biglerville, PA, or the Frost Insect Museum at Pennsylvania State University, University Park, PA.

### 2.3 Data and statistical analyses

All analyses were conducted with R version 4.0.3. To explore how thorough our sampling of bee biodiversity was we created a species accumulation curve based on the number of individuals sampled. We did this using the “specaccum” function in the vegan package (Oksanen et al., 2013).

We calculated biodiversity metrics for each of our 8 sampling locations using the combined data from both traps at each site. For month analyses (within years), we calculated biodiversity metrics for each month and then averaged across all years. We then averaged together sites closer than 900 m resulting in 4 replicates. Therefore, the interpretation of each replicate is the average biodiversity value per site for a single month. For year analyses (across years), we first summed data across all months, calculated metrics for each year, and then averaged to get 4 replicates per year. The interpretation of these data is then the total biodiversity value per site for a single year.

Using community abundance data, we measured total abundance, richness, and diversity (inverse Simpson's) within and across years using the “specnumber” and “diversity” functions in the vegan package (Oksanen et al., 2013). We also used community abundance data to measure differences in community composition using a Bray–Curtis dissimilarity matrix. To measure phylogenetic structure, we use a genus‐level molecular phylogeny from Hedtke et al. (2013). We made the phylogeny ultrametric with the “force.ultrametric” function in the phytools package (Revell, 2012) using the non‐Negative Least‐squares method. We then amended our species below the genus‐level using the “genus.to.species.tree” function which creates bifurcating subtrees among species in each genus and then binds them at a random place along the terminal edge. With this approach, the relationships among species below the genus level are created at random. We only had to drop 2 species from these analyses because their genera (*Cemolobus*, *Triepeolus*) were not in the tree, which only had 7 and 2 individuals in the dataset, respectively.

Using the community data and this phylogeny, we calculated mean pairwise distance (MPD) among all taxa. This metric is a measure of the average evolutionary distance between all pairs of species in a community and was calculated with the “mpd.query” function in the PhyloMeasures package (Tsirogiannis & Sandel, 2016). Because raw values of MPD can be impacted by species richness of samples, we used a standardized effect size of MPD that is based on the difference between the observed measure of MPD and a random null model of a community with the same number of species (Tsirogiannis & Sandel, 2016). This value is also standardized by variance making the metric an effect size in standard deviation units. Negative values of this metric indicate a community is more clustered (less average evolutionary distance among pairs of species) than a random community with the same richness, and positive values indicate a more even community. The resolution of phylogenies below the species level has little‐to‐no impact on MPD calculations. Qian and Jin (2021) found that MPD values are nearly identical when calculated with a genus‐level phylogeny with species amended (like ours) compared to a fully resolved phylogenetic tree. And in our case, repeating the process of randomly adding species below the genus level resulted in nearly perfectly correlated measures of MPD (*r* = 0.99). However, trees without species‐level resolution do not provide reliable estimates of phylogenetic signals of species' traits (Davies et al., 2012), so we did not include those analyses in this study.

We modeled the changes in bee biodiversity within and across years by fitting general additive models using the “gam” function in the mgcv package (Wood, 2017). These allowed for nonlinear fits to the data. For all tests, we used 5 knots which allowed for sufficient curviness to represent observed patterns and to produce linear relationships between observed and fitted values. The amount of variation explained by these models was typically similar to ANOVA models fit to the same data. We report percent change effect sizes among extreme values of months and years as the difference in means between a pair of values (e.g., the mean from April minus the mean for August) then divide that difference by the overall mean for that variable. This gives a standardized effect size that can be compared among variables. We used perMANOVA to test differences in composition among groups using the “escalc” function and visualized results with nonmetric multidimensional scaling fit with the “metaMDS” function, both from the vegan package (Oksanen et al., 2013).

To assess how phenological patterns differ among bee families and species, we calculated a metric for seasonality (time of year when the species showed the highest abundance) and phenological breadth (a measure of the amount of time bees are active as adults). Phenology measures were calculated for species with 30 or more individuals (a total of 40 species) as this is enough to reliably estimate phenological breadth (Bartomeus et al., 2013). We measured seasonality as the median day of year (Julian date) of capture across individuals collected for a species. Our measure of breadth was the difference between the 10th and 90th percentile of capture dates. To control for the different numbers of individuals across species, we randomly drew 30 data points for each species repeated 500 times. For each of these subsamples, we calculated the seasonality and breadth statistics and then averaged those 500 values to get our final statistics (Bartomeus et al., 2013).

To evaluate how bee families and species differ in their changes in abundance over time, we calculated a metric of change over time for each species as the slope of the linear relationship between abundance and year. We standardized abundance data for each species to have a mean of 0 and a standard deviation of 1 to allow comparisons across species. We multiplied the model coefficients by five (the change in years in our sampling) to get a predicted level of change in standard deviation units over the course of the study. We also tested if species‐level traits predicted species' model‐predicted change in abundance over time. We included two traits determined by our data, phenological breadth, seasonality, and 4 other traits gathered from the literature including: body length, social vs. solitary, specialized vs. generalist diet, and below ground versus above ground nesting. These natural history traits have been used in other studies to help explain changes in relative abundance over time (Bartomeus et al., 2013) and responses to environmental change (Hamblin et al., 2018; Pardee et al., 2022). We tested the relationship between these traits and change in abundance over time by fitting phylogenetic generalized linear models for each trait with a Brownian motion model of trait evolution. We used the “pgls” function in the caper package (Orme et al., 2018). We also fit linear models that did not account for evolutionary relationships among species. For simplicity we fit individual models for each predictor variable, but multiple regression models including all variables at once had similar results.

## 3 RESULTS

### 3.1 Description of biodiversity

**FIGURE 2:**
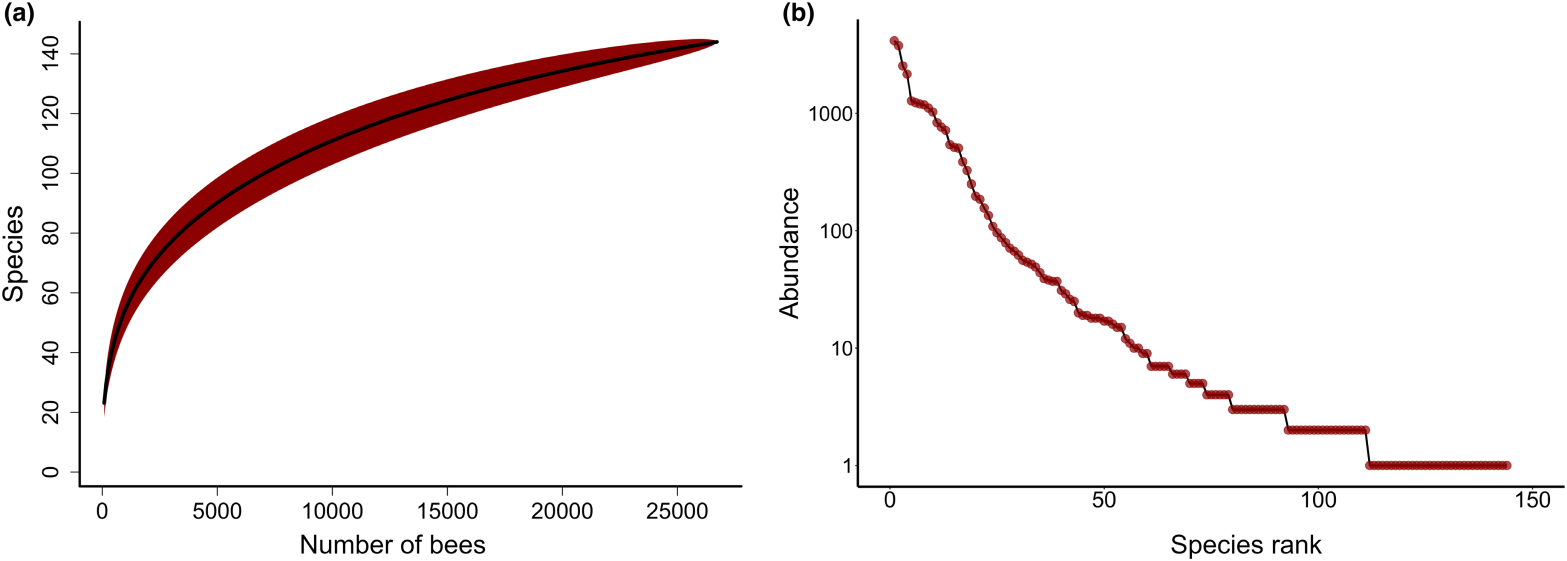
Species accumulation and rank abundance curves. (a) Species accumulation curve shows how the average number of species detected increases with the total number of bees collected. The flattening of the curve suggests that most, but not all, of bee biodiversity in the region is represented in our collections. (b) Rank abundance curve shows the number of individuals collected for each species ranked from highest to lowest, note the log *y*‐axis. In our dataset of over 26,000 bees, only 10 species had over 1000 individuals while over half of the species had 5 or fewer individuals.

Our final dataset included 26,716 individual bees representing 5 bee families, 30 genera, and 144 species. See Turley et al. (2022) for the complete dataset and species list. We collected 33% of the total number of bee species that have been found in Pennsylvania (Kilpatrick et al., 2020). The species accumulation curve for these data shows a leveling off pattern but did not reach an asymptote (Figure 2a). The abundances among species show a typical rank abundance curve with a small number of very abundant species and many rare species (Figure 2b). Ten species had over 1000 individuals while over half of the species had 5 or fewer individuals. We captured by far the most individuals and species from the family Apidae (19,870 individuals and 47 species) followed by Halictidae (5942 and 33), Andrenidae (477 and 28), Megachilidae (383 and 30), and Colletidae (44 and 6).

### 3.2 Biodiversity changes within years

**FIGURE 3:**
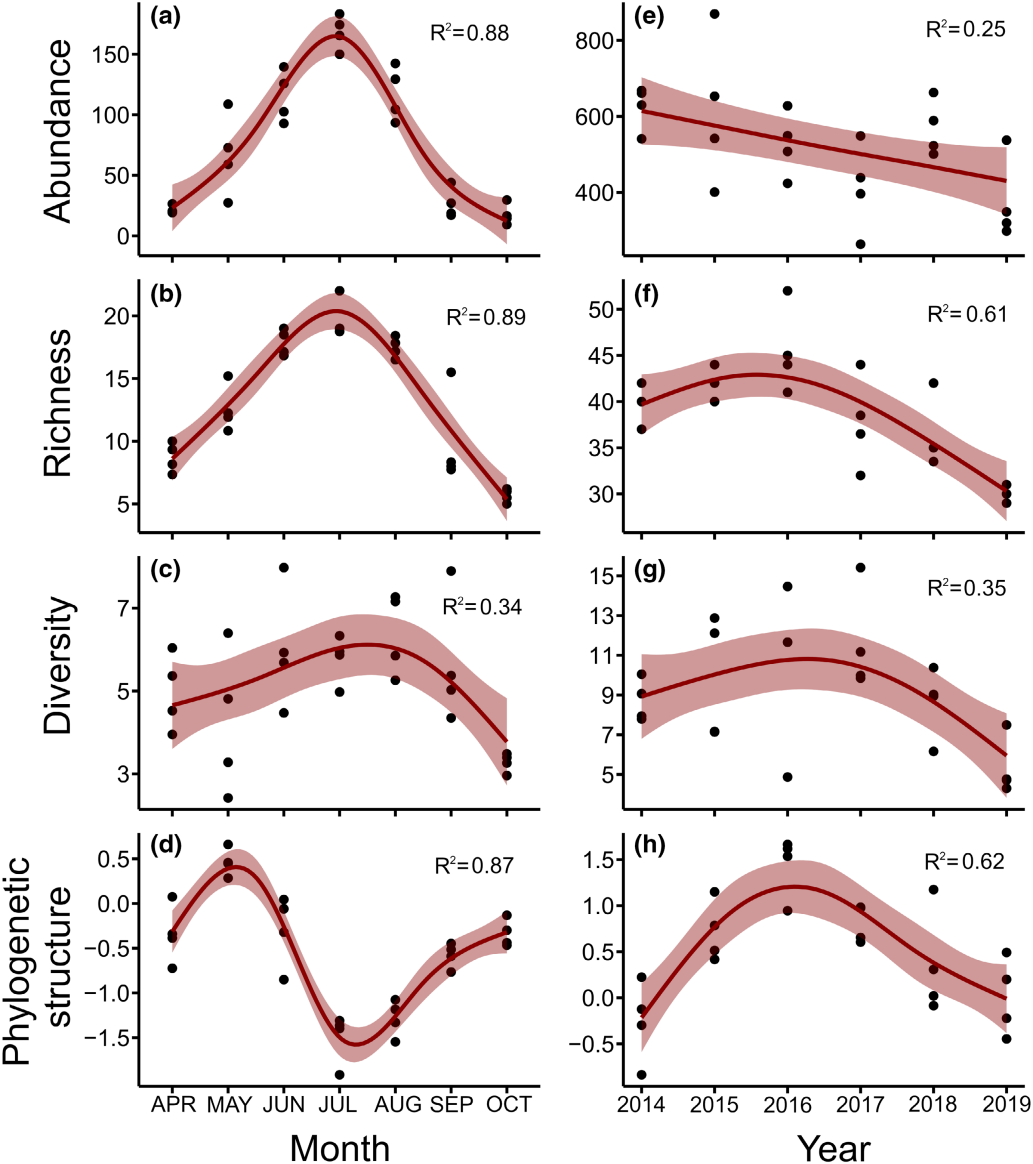
Patterns of bee biodiversity across months, and changes across years. All model relationships were highly significant (*p* < .002). Abundance (a, e) is the average number of bees collected per site. Richness (b, f) is the number of bee species per site. Diversity (c, g) is the inverse Simpson's diversity index. Phylogenetic structure (d, h) is the standardized effect size of mean pairwise distance (higher values are more even, and lower values are more clustered). Line fits and adjusted *R* ^2^ values are from general additive models and the shading represents 95% confidence intervals of the models.

We found very strong evidence for seasonal changes in all measures of biodiversity with month explaining an average of 74% of the variation in our models (Figure 3, Table 1). Abundance and richness showed a hump‐shaped pattern peaking in July. In our models, month explained nearly 90% of variation in abundance and richness. In April, we captured an average of 21 bees per site compared to 168 bees per site in July, an increase of 193% relative to the monthly average of 76 bees per site (Figure 3a). Similarly, average richness in April was 9 species per site and in July was 21 species, an increase of 89% (Figure 3b). Diversity also changed over time but less sharply than richness, and peaked in August instead of July (Figure 3c). Diversity increased by 41% between May and August and decreased by 60% from August to October (Figure 3c). Because diversity incorporates both richness and evenness, the weaker pattern in diversity compared to richness is a consequence of evenness having a pattern nearly opposite that seen in richness (*p* < .0001, *R* ^2^ = .68), highest in spring and fall and lowest in July.

**TABLE 1:** Model results for the effect of month (within‐year changes) and year (across‐year effects) on measures of biodiversity. Results for abundance, richness, diversity (inverse Simpson's), and phylogenetic structure (mean pairwise distance) are from generalized additive models and the test statistics are t‐values. Community composition results are from a perMANOVA and the test statistics are *F*‐values.

Month effects				Year effects			
Response variable	Test stat.	*p*	*R* ^2^	Response variable	Test stat.	*p*	*R* ^2^
Abundance	20.01	<.001	.88	Abundance	20.71	<.001	.25
Richness	38.70	<.001	.89	Richness	51.78	<.001	.61
Diversity	22.89	<.001	.34	Diversity	18.22	<.001	.35
Phylogenetic structure	14.16	<.001	.87	Phylogenetic structure	4.92	<.001	.62
Composition	6.21	<.001	.64	Composition	2.03	.002	.36

**FIGURE 4:**
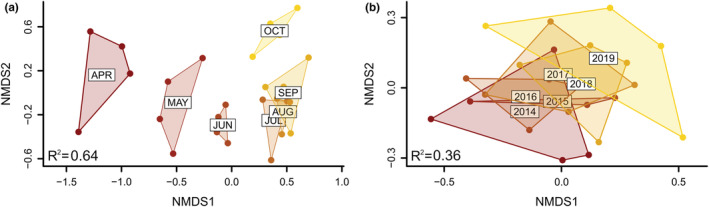
Effects of months (a) and years (b) on bee community composition. Both months and years have significant effects on bee composition but differences among months are larger than among years. Data are visualized using nonmetric multidimensional scaling on a Bray–Curtis dissimilarity matrix which includes species abundances. The *R* ^2^ values are the variance explained from perMANOVA models.

Phylogenetic structure also varied between months. Mean pairwise distance dropped (becoming more clustered) by 1.9 standard deviations between May and July and then increased (becoming more even) by 1.1 standard deviations between July and October. The months of April, June, August, and September had intermediate values (Figure 3d). We observed limited variation in phylogenetic structure between sites resulting in our model explaining 87% of the total variation (Table 1). Community composition varied substantially among months with our multivariate model explaining 64% of the variation in bee communities (Figure 4a, Table 1). Spring months (April–June) all had distinct bee communities. July through September had similar compositions which were themselves distinct from spring months and October (Figure 4a).

### 3.3 Biodiversity change across years

We found very strong evidence for biodiversity change over time between 2014 and 2019 with year explaining an average of 42% of variation across all biodiversity metrics (Figure 3, Table 1). The average abundance of bees captured per site in 2014 was 625 and in 2019 was 376, a decline of 48% (Figure 3e). Richness peaked in 2016 at 46 species per site and declined to 30 species in 2019, a change of 41% (Figure 3f). Like richness, diversity also declined after 2016, dropping by 59% between 2016 and 2019 (Figure 3g), though the model for diversity explained about half as much variation as the model for richness (Table 1).

Phylogenetic structure increased and decreased over time with the most clustered communities in 2014 and 2019, and the most even communities in 2016 (Figure 3h). Mean pairwise distance increased (became more even) by 1.8 standard deviations between 2014 and 2016, and then decreased (becoming more clustered) by 1.6 standard deviations between 2016 and 2019. Bee communities were quite stable across the first 3 years though they shifted slightly over time in the last 3 years (Figure 4b). Year explained 36% of the variation in community composition.

### 3.4 Species patterns in seasonality, phenological breadth, and change over time

**FIGURE 5:**
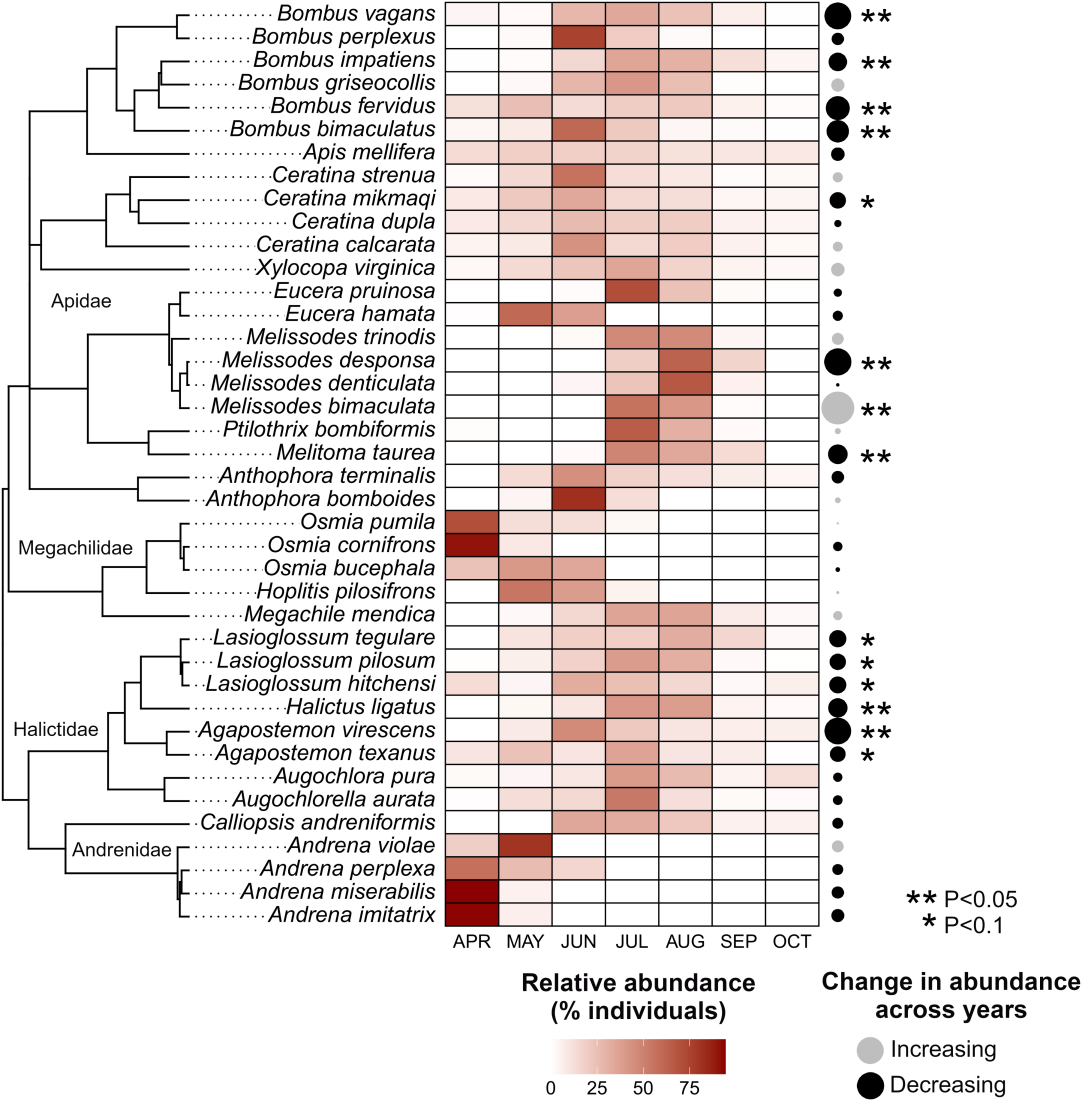
Species‐level phenological patterns and changes in abundance over time for 40 species with at least 30 individuals collected. The colored heat map shows the percentage of individuals captured for each species in each month, therefore a value of 100% would mean all individuals of that species were captured in that month. The black and gray points represent the positive or negative change in abundance over time. The size of the points are scaled by coefficients from linear models (i.e., slope of the relationship between year and abundance using standardized data). The phylogeny has our focal species amended (see methods) to a genus‐level tree by Hedtke et al. (2013).

**FIGURE 6:**
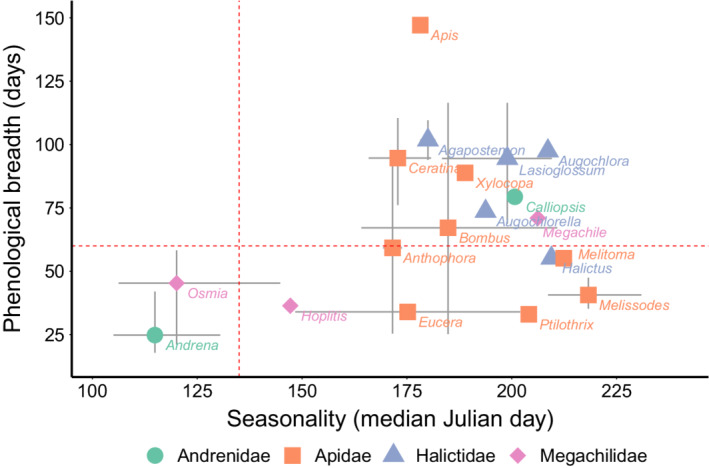
The distributions of both seasonality and phenological breadth among bee genera and families. Seasonality is the median Julian date in which each genus and species was captured across 6 years of continuous sampling. Phenological breadth is a measure of the length of time in which bees are active. Error bars show the highest and lowest values for species in each genus. Red dotted lines illustrate the conceptual idea of “phenological syndromes.” The bottom left quadrant are early emerging species with narrow phenological breadth. Species in the bottom right quadrant emerge in summer but still have narrow breadth. Species in the top right have wide phenological breadth and are most abundant in summer.

Looking across the 40 species for which we collected 30 or more individuals (Table A1), bee families varied significantly in seasonality (Figures 5 and 6, *F*_3,36_ = 10.91, *p* < .001). Species in the families Megachilidae and Andrenidae were collected an average of 54 days earlier than species in the families Apidae and Halictidae (Tukey tests, *p* < .006). In the family Megachilidae, *Osmia* species and *Hoplitis pilosifrons* were among the earliest emerging species, while *Megachile mendica* was most active in July and August (Figure 5). In the Andrenidae, all *Andrena* species were most active in April and May, but *Calliopsis andreniformis* was most abundant in July. Among species in the family Apidae, *Eucera hamata* was the only species with peak abundance in May. Species in the genera *Anthophora*, *Ceratina*, and *Bombus* were most active in June, though there is some variation among species within those genera. Other species in the family Apidae, including those in the genera *Ptilothrix*, *Melitoma*, and *Melissodes*, as well as *Eucera* (*Peponapis*) *pruinosa*, peaked in July and August (Figure 5). Most species in the family Halictidae were most abundant in July and August, though two *Agapostemon* species were active earlier (Figures 5 and 6).

Phenological breadth varied among families (Figures 5 and 6, *F*_3,36_ = 3.9, *p* = .02). Species in the family Andrenidae had the narrowest breadth at 35 days on average, and species in the Halictidae were active the longest at 89 days on average (Tukey test, *p* = .02). The families Megachilidae (49 days) and Apidae (66 days) showed intermediate breadth. In the Andrenidae, all *Andrena* species had a breadth of less than 42 days, but *Calliopsis andreniformis* had a breadth of 79 days. In the Megachilidae, *Osmia* species' breadths range from 21 to 58 days while *Megachile mendica* had a breadth of 71 days. Species in the Halictidae consistently had a wide phenological breadth, greater than 75 days, though *Halictus ligatus* had a relatively narrow window of 55 days. We split species in the Apidae into two groups, each consisting of related clades, that varied significantly in phenological breadth (*t*‐test, *t* = −3.7, df = 18.3, *p* = .002). Species in the genera *Bombus*, *Apis*, *Ceratina*, and *Xylocopa* had an average breadth of 85 days, while species in the genera *Eucera*, *Melissodes*, *Ptilothrix*, *Melitoma*, and *Anthophora* had an average breadth of 44 days (Figures 5 and 6).

We observed substantial species‐level variation in the changes in abundance across years (Figure 5, Table A1). We detected little‐to‐no change in abundance for 26 species (*p* > .1), 5 species showed weak evidence for decline (*p* < .1), 8 more showed moderate to strong evidence for decline (*p* < .05), and we found strong evidence for increase in 1 species (*p* < .01). While bee family was not a significant predictor of changes in abundance (*F*_3,36_ = 1.813, *p* = .16), we observed some patterns among families and genera. Species in the families Megachilidae and Andrenidae were all stable. Other families showed mixed trends. For example, among species in the family Apidae, all carpenter bee species in the genera *Xylocopa* and *Ceratina* were stable except for *Ceratina mikmaqi*, which declined by 1 standard deviation unit (SD) between 2014 and 2019. Six out of eight species in the Halictidae showed evidence for declines, most species by about 1 SD, but *Agapostemon virescens* declined by 1.7 SD. Four species in the genus *Bombus* showed pronounced declines ranging between 1.2 and 1.8 SD while two *Bombus* species were stable. Similarly, *Melitoma taurea* declined by 1.3 SD. We saw radical variation among species in tribe Eucerini (Apidae): some species stable over time (*Eucera* and some *Melissodes*), *Melissodes desponsa* had the biggest decline we observed (1.8 SD), and *Melissodes bimaculatus* increased by 2.2 SD, which was the only significant increase and also the largest magnitude of change we found.

Natural history traits did not predict variation in species' changes in abundance over time for our 40 focal species (Table A2). Natural history traits quantified from this study (seasonality and phenological breadth) and factors gleaned from the literature (body length, sociality, below‐ground nesting, diet specialization) showed no significant relationships with change over time in phylogenetic linear models (Table A2). In models that did not control for phylogeny, we found that social species had a 0.7 SD greater decline than solitary species (*p* = .02).

## 4 DISCUSSION

With 6 years of continuous sampling with passive traps, we collected over 26,000 bees and 144 species. The leveling off of the species accumulation curve suggests we captured most, but not all, of bee biodiversity in the system (Figure 2a). The inability to fully document biodiversity (i.e., reach an asymptote in the species accumulation curve) is typical for other extensive bee monitoring efforts (Russo et al., 2015; Wilson et al., 2008) and species‐rich invertebrate communities more generally because there are many rare species (Gotelli & Colwell, 2001). Also, passive traps are not attractive to all species so our results should be interpreted as a subset of total bee species in the system which are attracted to Blue Vane traps (Prendergast et al., 2020).

We found that all measures of community biodiversity varied dramatically within years and across years (Figures 3 and 4). Abundance, richness, and diversity all peaked in July, though diversity to a lesser extent. These results are congruent with previous long‐term studies of bee communities. Leong et al. (2016) repeatedly sampled bee communities across several habitat types in Central California, USA, and also found peaks in abundance and richness, but in May rather than in July. In contrast, Neave et al. (2020) studied bee phenology at a single site in South‐Eastern Australia and found peak abundance in spring, though patterns varied greatly from year to year and appeared to be affected by rainfall patterns. We found that community composition also varied greatly within years with the largest shifts observed in spring then becoming much more stable between July and September (Figure 4). The changes we found in community composition closely mirrors results of Kammerer et al. (2021) who also found that bee communities sampled with passive traps in nearby Maryland were distinct in each of the first 3 months of spring then similar for the rest of the year. Our study is one of the few that have studied phylogenetic diversity over time, and we found that phylogenetic structure was most even in spring (May) and became most clustered in summer.

We also found that all measures of biodiversity changed across years (Figures 3 and 4). These changes in diversity metrics and composition were partially driven by 13 species that declined in abundance over time, which were dispersed across the bee evolutionary tree (Figure 5). The reasons for these changes over time are not clear. While habitat loss, land‐use changes, and pesticide use all likely impact bee communities in this system, these were all relatively unchanging over the course of this study (Biddinger et al., 2018). Changes in the floral resources of the flower strips where we sampled could have been a factor since they likely experience a reduction of plant diversity over time, as is typical in restored grasslands (Sluis, 2002). Similar studies that have sampled bee communities over time using standardized sampling in North America have found variable results related to changes in abundance. Onuferko et al. (2018) found declines in abundance and richness over 10 years in undisturbed grasslands. Graham et al. (2021) found increases and decreases in bee abundance in blueberry fields potentially caused by changes in precipitation across 15 years. Surveys of bumble bees for 8 years in the Rocky Mountains showed no directional trends in abundance (Ogilvie et al., 2017) and in Coastal California, there were declines in abundance across 10 years, potentially caused by competition with honey bees and climate‐driven changes in floral resources (Thomson, 2016). Finally, Aldercotte et al. (2022) found declines in abundance of visitors to watermelon flowers over 8 years. In some studies, natural history traits helped explain changes in bee abundance over time or responses to environmental fluctuations (Bartomeus et al., 2013; Hamblin et al., 2018). For example, Pardee et al. (2022) found that bee body size and nesting strategy impacted species' responses to changing temperature. We found that 6 commonly studied natural history traits did not predict changes in abundance when controlling for phylogeny. There was a small effect of social species declining more than solitary species when not controlling for phylogeny, but this was likely driven only by declines in bumble bees.

Insect monitoring efforts can find evidence for directional increases and decreases in abundance over time that are actually the result of sampling starting or ending at high or low abundance years (Aldercotte et al., 2022; Didham et al., 2020; Fournier et al., 2019). Because of the complicating effects of year‐to‐year variability, 10 or more years of monitoring may be needed to come to strong conclusions about trends in population dynamics (Didham et al., 2020; Fournier et al., 2019). Two pollinator monitoring efforts which meet this criteria are Herrera (2019) who made standardized observations of pollinators in Spain for 20 years and Roubik et al. (2021) who monitored orchid bees in Panama for 40 years. Both studies took place in large undisturbed natural habitats and both concluded pollinator populations were mostly stable. Given the lack of other similar monitoring efforts in urban or agricultural areas, it is not clear if the stability seen in these two studies is because these are in high‐quality habitats, or because the long‐term monitoring helps buffer against spurious conclusions of pollinator declines. Because our sampling was for 6 years, it is difficult to know if the declines in abundance and community biodiversity we observed are representative of a larger trend or a result of taking a snapshot of on‐going yearly fluctuations (Aldercotte et al., 2022). For example, richness, diversity, and phylogenetic structure all reached a peak in 2016, so it is possible that something about the environmental conditions in 2015 or 2016 increased biodiversity compared to the long‐term mean, and declines we observed were just a product of returning to normal. One other factor to consider in interpreting biodiversity changes over time is the coefficient of variation (CV) from year‐to‐year, as greater variability can reduce power to detect changes over time. Lebuhn et al. (2013) used data from 11 pollinator monitoring studies using pan traps and found that the CV for abundance and richness measures was about 40%, and another study using Blue Vane traps had CV of around 50% (Tronstad et al., 2022). In our data the CV for abundance was 25% and richness was 15%. So, while monitoring for several more years would make for more concrete conclusions about biodiversity changes, our sampling had above‐average power to detect them.

### 4.1 Insights from species‐level changes in abundance

Species across the bee evolutionary tree showed a wide variety of phenological patterns (changes in abundance within years). Among the 40 species for which we had sufficient data, we observed three general patterns, which could be called “phenological syndromes” (Figure 6). First, solitary species in the genera *Andrena* and *Osmia* emerged early in the year and had narrow breadth. Second, solitary ground‐nesting species in the tribe Eucerini, (*Melissodes* and *Eucera*), and other sister clades had narrow breadth but, in the summer, rather than in spring. The third group was composed of species with wide phenological breadth including the social and multivoltine species in the genera *Bombus*, *Apis*, *Xylocopa*, and *Ceratina*, and nearly all the sweat bees (family Halictidae). Monitoring of species that represent these different phenological syndromes is important because they provide unique ecological functions (Ogilvie & Forrest, 2017). For example, many of the early emerging bee species are of critical importance for early flowering plants such as spring ephemeral wildflowers, and these interactions may be particularly sensitive to disruptions from climate change (Kudo & Ida, 2013). And many crops such as apples and blueberries rely on pollination by early emerging wild bees (Biddinger et al., 2018; Grab et al., 2019; Isaacs & Kirk, 2010; Reilly et al., 2020).

Similar to other studies, we found that 33% of species had at least some evidence of declines while only 3% increased, and 65% percent showed no changes over time. For example, a study using museum records of 187 bee species in eastern North America found significant decreases in the relative number of samples in collections for 29% of species and increases for 27% of species (Bartomeus et al., 2013). Similarly, 38% of nonparasitic bumble‐bee species in the United Kingdom show clear signs of decline (Williams & Osborne, 2009). While it is possible that we could have had more power to detect changes in rare species with more thorough sampling, we found significant changes among species with a wide variety of abundances (min = 37, max = 3774, mean = 894) and there was no correlation between species' total abundance and amount of predicted change (*r* = .18, *p* = .26). The amount of year‐to‐year variation in species abundance could also influence the power to detect changes over time (Lebuhn et al., 2013). Species' yearly coefficient of variation (CV) had a mean of 92% (min = 43, max = 182, SD = 37). Species with significant changes over time had a broad range of variation (min = 43, max = 166, mean = 87) and CV was not correlated with predicted change in abundance (*r* = .1, *p* = .56). Therefore, this suggests that neither sample size or excess variation limited our ability to detect changes over time and that our finding of 65% of species being stable is robust.

We did not find that bee family or natural history traits were significant predictors of which bees were stable or declining. While there were some clades that were more prone to declines than others, notably bumble bees (*Bombus*) and sweat bees (Halictidae), we do not have any specific evidence to explain why some groups declined while others did not. In one case, 2 closely related longhorn bees (genus *Melissodes*) showed large changes in abundance in opposite directions while the other 2 *Melissodes* species were stable. The increasing species (*M. bimaculata*) is a very common species and a generalist, while the decreasing species (*M. desponsa*) is specialized, feeding primarily on thistles. The other two stable *Melissodes* species are also specialist. Other studies have found that diet specialization in bumble bees is a good predictor of changes in geographic ranges over time, with specialists more likely to decline (Wood et al., 2019). So, while in our study diet specialization did not predict changes over time across all species, it is still possible that the generalist diet of *M. bimaculata* played a role in their large spike in abundance in the last 2 years of the study. Focused studies that track changes in abundance of flowering host plants may help in providing mechanisms to which species are increasing or decreasing (Herrera, 2019; Thomson, 2016). Understanding which adaptations or life history traits are associated with population increases or decreases over time in some cases could be a better approach to explain species' changes in abundance over time (e.g., Stemkovski et al., 2020; Williams et al., 2009). However, given that a suite of natural history traits was not associated with the species‐level changes we observed (Table A2), it may take more investigation into environmental variable to understand what mechanism may be driving species' population changes over time in our system (Hamblin et al., 2018; Ogilvie et al., 2017; Pardee et al., 2022; Stemkovski et al., 2020).

### 4.2 Insights from multiple measures of community biodiversity

Our thorough collections of bees throughout the seasons, and measurements of communities using a variety of metrics, highlighted the unique biodiversity of bee communities in the spring. We were also able to see four ways in which different biodiversity metrics resulted in varying conclusions about the community changes across the year. First, the simplest measures of abundance and richness suggested that biodiversity in the spring is low. Second, by contrast, diversity (measured as inverse Simpson's) was similar in April and May as it was in July and August despite huge differences in richness. This relative elevation of diversity in the spring was a consequence of greater evenness, or more equal abundances among species which results in quicker accumulation of species. Similarly, the total amount of spring species captured across all sites and all years (as opposed to the site‐level averages for each year results presented in Figure 2) was also high in spring despite the low abundances. Using rarefaction to standardize the number of individuals collected, we detected a total of 58 species per 900 individuals in April compared to 40 species per 900 individuals in July. Third, we found that May had the most phylogenetically even (overdispersed) communities which became much more clustered by July. A nonmechanistic interpretation is that in May, spring bees (largely from Andrenidae and Megachilidae) and some summer bees (mostly in Apidae and Halictidae; Figure 5) were both active resulting in long branch lengths between pairs of species. This parallels results by Ramírez et al. (2015) who found that orchid bee communities in Colombia were much more phylogenetically even in the transition period between wet and dry seasons. Fourth, community composition results were able to show the quick turnover of species in the spring with April, May, and June all being distinct communities. These unique aspects of spring biodiversity would be completely missed by looking at only abundance and richness measures and not diversity, phylogenetic structure, and composition. This suggests that studies seeking to understand the phenological changes of bee communities and the impacts of environmental change on spring bees need to have robust sampling and multiple measures of bee biodiversity.

Repeated measures of bee communities across years suggested a loss of community biodiversity over time, though the patterns of declines depend on which metric you look at (Figure 3). From a bee monitoring and conservation perspective, changes in abundance, richness, and diversity are easy to interpret. In most cases, decreases in these metrics suggest that some environmental degradation or changes in species interactions are causing losses of biodiversity. However, metrics like composition, and phylogenetic structure are harder to interpret without a reference point but can reveal changes not seen in simpler measures (Nerlekar & Veldman, 2020; Tucker et al., 2017). For example, Tonietto et al. (2017) found that old fields, restored prairies, and remnant prairies all had the same abundance and diversity of bees but community compositions were different. And similarly, Frishkoff et al. (2014) found that one type of agricultural land‐use did not change bird richness, but it did lead to more phylogenetically clustered communities compared to forest reserves. Going forward, more long‐term bee monitoring studies are needed to determine if biodiversity measures like composition and phylogenetic structure provide unique and useful information for conservation efforts.

### 4.3 Implications for bee monitoring

There are a variety of bee monitoring approaches that range from standardized and repeated collections of bees with detailed taxonomic identification to visual observations of broad taxonomic groups that involve participation from the public. There are pros and cons to studies using methods on both ends of this spectrum (Woodard et al., 2020). Our approach involved continuous collecting using passive Blue Vane traps and species‐level identification of all bees. The sampling throughout the year gave us the ability to quantify seasonal changes in biodiversity with fine resolution. The large number of bees collected translated into data availability for 40 species to characterize phenological patterns. And the standardized sampling over several years allowed us to quantify changes in abundance over time, something many studies have limitations with (Portman et al., 2020). However, it is important to highlight that studies using passive trapping need to be interpreted with caution as the data do not reflect true population sizes (Briggs et al., 2022; Portman et al., 2020). This is because some species are more attracted to traps than others and because trapping results are impacted by context (Kuhlman et al., 2021). While our data may not reflect the absolute abundance of species in the wild, it does show that standardized passive trapping is effective at measuring relative changes within and across years. Overall, the intensive type of monitoring of our study is a good approach to answer questions about community biodiversity change and the unique population dynamics across many co‐occurring species.

Our sampling approach has several constraints for its implementation on large‐scale monitoring projects that aim to detect bee declines. First, collecting, processing, and databasing large numbers of bees is labor‐intensive and taxonomic identification requires specialized skills and expertise. This makes specimen curation and identification potentially untenable and impractical for large‐scale projects. Second, tens of thousands of bees were killed in the sampling. Concerns have been raised that sampling many bees with Blue Vane traps could cause declines in some species (Gibbs et al., 2017). While we did not estimate how our collections impacted populations, the lack of correlation between the number of individuals captured and species changes in abundance over time (*r* = .18, *p* = .26) provides at least some evidence that this was not the case in our study. Third, passive traps have inherent biases in the species they collect and these biases impact biodiversity metrics. While collections with other techniques would have resulted in different levels of observed biodiversity, we know from our system that Blue Vane traps provide thorough sample of the whole bee community (Joshi et al., 2015). And finally, our collections are only from one relatively small area (Figure 1). Given the local nature of our dataset, the observed changes within and across years could be unique to our study area. However, similar phenological patterns and declines have been found in other studies (Bartomeus et al., 2013; Graham et al., 2021; Kammerer et al., 2021; Leong et al., 2016).

## 5 CONCLUSIONS

Concerns about the ecological consequences of changes in bee biodiversity are leading to increased recognition of the importance of wild bee conservation and the enhancement of wild bees in agricultural systems (Biddinger et al., 2018; Isaacs & Kirk, 2010; Reilly et al., 2020). But, wild bee communities are diverse and dynamic, and little is known about what species or groups have the greatest conservation needs. Our intensive sampling across 6 years shows that bee communities vary greatly from month to month for all measures of biodiversity. For monitoring efforts to capture the full breadth of bee biodiversity, it is important to sample bees across all seasons, especially during spring when communities turnover rapidly. We also found evidence for changes across the 6 years of our study with all biodiversity metrics declining in the last 3 years. We detected declines in abundance in 33% of the species. However, it will take additional years of monitoring to determine if these changes over time are part of a larger trend or a consequence of year‐to‐year fluctuations (Didham et al., 2020). Neither evolutionary history nor species' natural history traits explained species‐level population dynamics. We recommend that future monitoring efforts focus on species‐level sampling for multiple co‐occurring species to understand population‐level processes driving changes over time. These studies could be critical to identify species of conservation concern (Woodard et al., 2020).

## AUTHOR CONTRIBUTIONS

**Nash E Turley:** Formal analysis (lead); visualization (lead); writing – original draft (lead); writing – review and editing (equal). **David Biddinger:** Conceptualization (equal); funding acquisition (equal); investigation (lead); methodology (equal); writing – review and editing (equal). **Neelendra K Joshi:** Conceptualization (equal); investigation (equal); methodology (equal); writing – review and editing (equal). **Margarita M López‐Uribe:** Conceptualization (equal); funding acquisition (lead); writing – review and editing (lead).

## CONFLICT OF INTEREST

The authors declare no conflicts of interest.

## Supporting information


Appendix S1

## Data Availability

The complete datasets used for the analyses in this study are available on Dryad: https://doi.org/10.5061/dryad.9kd51c5mc.

## References

[ece39190-bib-0001] Aldercotte, A. H. , Simpson, D. T. , & Winfree, R. (2022). Crop visitation by wild bees declines over an 8‐year time series: A dramatic trend, or just dramatic between‐year variation? Insect Conservation and Diversity. 10.1111/icad.12589

[ece39190-bib-0002] Bartomeus, I. , Ascher, J. S. , Gibbs, J. , Danforth, B. N. , Wagner, D. L. , Hedtke, S. M. , & Winfree, R. (2013). Historical changes in northeastern US bee pollinators related to shared ecological traits. Proceedings of the National Academy of Sciences of the United States of America, 110(12), 4656–4660. 23487768 10.1073/pnas.1218503110 PMC3606985

[ece39190-bib-0003] Biddinger, D. J. , Rajotte, E. G. , & Joshi, N. K. (2018). Integrating pollinator health into tree fruit IPM—A case study of Pennsylvania apple production. In D. W. Roubik (Ed.), The pollination of cultivated plants: A compendium for practitioners (Vol. 1, pp. 69–83). Food and Agricultural Organization of the United Nations.

[ece39190-bib-0004] Biesmeijer, J. C. , Roberts, S. P. , Reemer, M. , Ohlemuller, R. , Edwards, M. , Peeters, T. , Schaffers, A. P. , Potts, S. G. , Kleukers, R. J. M. C. , Thomas, C. D. , & Settele, J. (2006). Parallel declines in pollinators and insect‐pollinated plants in Britain and The Netherlands. Science, 313(5785), 351–354. 16857940 10.1126/science.1127863

[ece39190-bib-0005] Briggs, E. L. , Baranski, C. , Münzer Schaetz, O. , Garrison, G. , Collazo, J. A. , & Youngsteadt, E. (2022). Estimating bee abundance: Can mark‐recapture methods validate common sampling protocols? Apidologie, 53(1), 1–24.

[ece39190-bib-0006] Burkle, L. A. , Marlin, J. C. , & Knight, T. M. (2013). Plant‐pollinator interactions over 120 years: Loss of species, co‐occurrence, and function. Science, 339(6127), 1611–1615. 23449999 10.1126/science.1232728

[ece39190-bib-0007] Cameron, S. A. , Lozier, J. D. , Strange, J. P. , Koch, J. B. , Cordes, N. , Solter, L. F. , & Griswold, T. L. (2011). Patterns of widespread decline in north American bumble bees. Proceedings of the National Academy of Sciences of the United States of America, 108(2), 662–667. 21199943 10.1073/pnas.1014743108 PMC3021065

[ece39190-bib-0008] Cane, J. (2021). Global warming, advancing bloom and evidence for pollinator plasticity from long‐term bee emergence monitoring. Insects, 12(5), 457. 34065667 10.3390/insects12050457 PMC8155920

[ece39190-bib-0009] Davies, T. J. , Kraft, N. J. , Salamin, N. , & Wolkovich, E. M. (2012). Incompletely resolved phylogenetic trees inflate estimates of phylogenetic conservatism. Ecology, 93(2), 242–247. 22624305 10.1890/11-1360.1

[ece39190-bib-0010] Didham, R. K. , Basset, Y. , Collins, C. M. , Leather, S. R. , Littlewood, N. A. , Menz, M. H. M. , Müller, J. , Packer, L. , Saunders, M. E. , Schönrogge, K. , Stewart, A. J. A. , Yanoviak, S. P. , & Hassall, C. (2020). Interpreting insect declines: Seven challenges and a way forward. Insect Conservation and Diversity, 13, 103–114. 10.1111/icad.12408

[ece39190-bib-0011] Fournier, A. M. , White, E. R. , & Heard, S. B. (2019). Site‐selection bias and apparent population declines in long‐term studies. Conservation Biology, 33(6), 1370–1379. 31210365 10.1111/cobi.13371

[ece39190-bib-0012] Frishkoff, L. O. , Karp, D. S. , M'Gonigle, L. K. , Mendenhall, C. D. , Zook, J. , Kremen, C. , Hadly, E. A. , & Daily, G. C. (2014). Loss of avian phylogenetic diversity in neotropical agricultural systems. Science, 345(6202), 1343–1346. 25214627 10.1126/science.1254610

[ece39190-bib-0013] Garibaldi, L. A. , Steffan‐Dewenter, I. , Winfree, R. , Aizen, M. A. , Bommarco, R. , Cunningham, S. A. , Kremen, C. , Carvalheiro, L. G. , Harder, L. D. , Afik, O. , & Bartomeus, I. (2013). Wild pollinators enhance fruit set of crops regardless of honey bee abundance. Science, 339(6127), 1608–1611. 23449997 10.1126/science.1230200

[ece39190-bib-0014] Gezon, Z. J. , Wyman, E. S. , Ascher, J. S. , Inouye, D. W. , & Irwin, R. E. (2015). The effect of repeated, lethal sampling on wild bee abundance and diversity. Methods in Ecology and Evolution, 6(9), 1044–1054.

[ece39190-bib-0015] Gibbs, J. , Joshi, N. K. , Wilson, J. K. , Rothwell, N. L. , Powers, K. , Haas, M. , Gut, L. , Biddinger, D. J. , & Isaacs, R. (2017). Does passive sampling accurately reflect the bee (Apoidea: Anthophila) communities pollinating apple and sour cherry orchards? Environmental Entomology, 46(3), 579–588. 28379550 10.1093/ee/nvx069

[ece39190-bib-0016] Gotelli, N. J. , Booher, D. B. , Urban, M. C. , Ulrich, W. , Suarez, A. V. , Skelly, D. K. , Russell, D. J. , Rowe, R. J. , Rothendler, M. , Rios, N. , Rehan, S. M. , Ni, G. , Moreau, C. S. , Magurran, A. E. , Jones, F. A. M. , Graves, G. R. , Fiera, C. , Burkhardt, U. , & Primack, R. B. (2021). Estimating species relative abundances from museum records. Methods in Ecology and Evolution, 1–13. 10.1111/2041-210X.13705

[ece39190-bib-0017] Gotelli, N. J. , & Colwell, R. K. (2001). Quantifying biodiversity: Procedures and pitfalls in the measurement and comparison of species richness. Ecology Letters, 4(4), 379–391.

[ece39190-bib-0018] Goulson, D. , Nicholls, E. , Botías, C. , & Rotheray, E. L. (2015). Bee declines driven by combined stress from parasites, pesticides, and lack of flowers. Science, 347(6229), 1255957. 25721506 10.1126/science.1255957

[ece39190-bib-0019] Grab, H. , Branstetter, M. G. , Amon, N. , Urban‐Mead, K. R. , Park, M. G. , Gibbs, J. , Blitzer, E. J. , Poveda, K. , Loeb, G. , & Danforth, B. N. (2019). Agriculturally dominated landscapes reduce bee phylogenetic diversity and pollination services. Science, 363(6424), 282–284. 30655441 10.1126/science.aat6016

[ece39190-bib-0020] Graham, K. K. , Gibbs, J. , Wilson, J. , May, E. , & Isaacs, R. (2021). Resampling of wild bees across fifteen years reveals variable species declines and recoveries after extreme weather. Agriculture, Ecosystems & Environment, 317, 107470.

[ece39190-bib-0021] Hamblin, A. L. , Youngsteadt, E. , & Frank, S. D. (2018). Wild bee abundance declines with urban warming, regardless of floral density. Urban Ecosystem, 21(3), 419–428.

[ece39190-bib-0022] Hedtke, S. M. , Patiny, S. , & Danforth, B. N. (2013). The bee tree of life: A supermatrix approach to apoid phylogeny and biogeography. BMC Evolutionary Biology, 13(1), 1–13. 23822725 10.1186/1471-2148-13-138 PMC3706286

[ece39190-bib-0023] Heithaus, E. R. (1979). Community structure of neotropical flower visiting bees and wasps: Diversity and phenology. Ecology, 60(1), 190–202.

[ece39190-bib-0024] Herrera, C. M. (2019). Complex long‐term dynamics of pollinator abundance in undisturbed Mediterranean montane habitats over two decades. Ecological Monographs, 89(1), e01338.

[ece39190-bib-0025] Isaacs, R. , & Kirk, A. K. (2010). Pollination services provided to small and large highbush blueberry fields by wild and managed bees. Journal of Applied Ecology, 47(4), 841–849.

[ece39190-bib-0026] Iserbyt, S. , & Rasmont, P. (2012). The effect of climatic variation on abundance and diversity of bumblebees: A ten‐year survey in a mountain hotspot. Annales de la Société entomologique de France, 48(3–4), 261–273.

[ece39190-bib-0027] Joshi, N. K. , Leslie, T. , Rajotte, E. G. , Kammerer, M. A. , Otieno, M. , & Biddinger, D. J. (2015). Comparative trapping efficiency to characterize bee abundance, diversity, and community composition in apple orchards. Annals of the Entomological Society of America, 108(5), 785–799.

[ece39190-bib-0028] Jost, L. (2006). Entropy and diversity. Oikos, 113(2), 363–375.

[ece39190-bib-0029] Kammerer, M. , Goslee, S. C. , Douglas, M. R. , Tooker, J. F. , & Grozinger, C. M. (2021). Wild bees as winners and losers: Relative impacts of landscape composition, quality, and climate. Global Change Biology, 27(6), 1250–1265. 33433964 10.1111/gcb.15485 PMC7986353

[ece39190-bib-0030] Kammerer, M. A. , Biddinger, D. J. , Rajotte, E. G. , & Mortensen, D. A. (2016). Local plant diversity across multiple habitats supports a diverse wild bee community in Pennsylvania apple orchards. Environmental Entomology, 45(1), 32–38. 26385933 10.1093/ee/nvv147

[ece39190-bib-0031] Kilpatrick, S. K. , Gibbs, J. , Mikulas, M. M. , Spichiger, S. E. , Ostiguy, N. , Biddinger, D. J. , & Lopez‐Uribe, M. M. (2020). An updated checklist of the bees (Hymenoptera, Apoidea, Anthophila) of Pennsylvania, United States of America. Journal of Hymenoptera Research, 77, 1–86.

[ece39190-bib-0032] Klein, A. M. , Vaissiere, B. E. , Cane, J. H. , Steffan‐Dewenter, I. , Cunningham, S. A. , Kremen, C. , & Tscharntke, T. (2007). Importance of pollinators in changing landscapes for world crops. Proceedings of the Royal Society B: Biological Sciences, 274(1608), 303–313. 10.1098/rspb.2006.3721 PMC1702377 17164193

[ece39190-bib-0033] Koh, I. , Lonsdorf, E. V. , Williams, N. M. , Brittain, C. , Isaacs, R. , Gibbs, J. , & Ricketts, T. H. (2016). Modeling the status, trends, and impacts of wild bee abundance in the United States. Proceedings of the National Academy of Sciences of the United States of America, 113(1), 140–145. 26699460 10.1073/pnas.1517685113 PMC4711882

[ece39190-bib-0034] Kudo, G. , & Ida, T. Y. (2013). Early onset of spring increases the phenological mismatch between plants and pollinators. Ecology, 94(10), 2311–2320. 24358716 10.1890/12-2003.1

[ece39190-bib-0035] Kuhlman, M. P. , Burrows, S. , Mummey, D. L. , Ramsey, P. W. , & Hahn, P. G. (2021). Relative bee abundance varies by collection method and flowering richness: Implications for understanding patterns in bee community data. Ecological Solutions and Evidence, 2(2), e12071.

[ece39190-bib-0036] Lebuhn, G. , Droege, S. , Connor, E. F. , Gemmill‐Herren, B. , Potts, S. G. , Minckley, R. L. , Griswold, T. , Jean, R. , Kula, E. , Roubik, D. W. , & Cane, J. (2013). Detecting insect pollinator declines on regional and global scales. Conservation Biology, 27(1), 113–120. 23240651 10.1111/j.1523-1739.2012.01962.x

[ece39190-bib-0037] Leong, M. , Ponisio, L. C. , Kremen, C. , Thorp, R. W. , & Roderick, G. K. (2016). Temporal dynamics influenced by global change: Bee community phenology in urban, agricultural, and natural landscapes. Global Change Biology, 22(3), 1046–1053. 26663622 10.1111/gcb.13141

[ece39190-bib-0038] Mallinger, R. E. , Gaines‐Day, H. R. , & Gratton, C. (2017). Do managed bees have negative effects on wild bees? A systematic review of the literature. PloS One, 12(12), e0189268. 29220412 10.1371/journal.pone.0189268 PMC5722319

[ece39190-bib-0039] Martins, A. C. , Gonçalves, R. B. , & Melo, G. A. (2013). Changes in wild bee fauna of a grassland in Brazil reveal negative effects associated with growing urbanization during the last 40 years. Zoologia (Curitiba), 30(2), 157–176.

[ece39190-bib-0040] Mathiasson, M. E. , & Rehan, S. M. (2019). Status changes in the wild bees of North‐Eastern North America over 125 years revealed through museum specimens. Insect Conservation and Diversity, 12(4), 278–288.

[ece39190-bib-0041] Michener, C. D. (2000). The bees of the world. The John Hopkins University Press.

[ece39190-bib-0042] Michener, C. D. , McGinley, R. J. , & Danforth, B. N. (1994). The bee genera of north and Central America (Hymenoptera:Apoidea). Smithsonian Institution Press.

[ece39190-bib-0043] Mitchell, T. B. (1960). Bees of the eastern United States volume 1. Technical Bulletin North Carolina Agricultural Experiment Station, 141, 1–538.

[ece39190-bib-0044] Mitchell, T. B. (1962). Bees of the eastern United States volume 2. Technical Bulletin North Carolina Agricultural Experiment Station., 152, 1–557.

[ece39190-bib-0045] Neave, M. J. , Brown, J. , Batley, M. , Rao, S. , & Cunningham, S. A. (2020). Phenology of a bee (Hymenoptera: Apoidea) community over a 10 year period in South‐Eastern Australia. Austral Entomology, 59(3), 602–611.

[ece39190-bib-0046] Neff, J. L. , & Simpson, B. B. (1993). Bees, pollination systems and plant diversity. In J. LaSalle & I. D. Gauld (Eds.), Hymenoptera and biodiversity (pp. 143–167). CAB International.

[ece39190-bib-0047] Nerlekar, A. N. , & Veldman, J. W. (2020). High plant diversity and slow assembly of old‐growth grasslands. Proceedings of the National Academy of Sciences of the United States of America, 117(31), 18550–18556. 32675246 10.1073/pnas.1922266117 PMC7414179

[ece39190-bib-0048] NRCS . (2011). Pollinator habitat‐conservation reserve program job sheet, CP42. P1‐5 . https://www.nrcs.usda.gov/Internet/FSE_DOCUMENTS/nrcs142p2_017524.pdf

[ece39190-bib-0049] Ogilvie, J. E. , & Forrest, J. R. (2017). Interactions between bee foraging and floral resource phenology shape bee populations and communities. Current Opinion in Insect Science, 21, 75–82. 28822493 10.1016/j.cois.2017.05.015

[ece39190-bib-0050] Ogilvie, J. E. , Griffin, S. R. , Gezon, Z. J. , Inouye, B. D. , Underwood, N. , Inouye, D. W. , & Irwin, R. E. (2017). Interannual bumble bee abundance is driven by indirect climate effects on floral resource phenology. Ecology Letters, 20(12), 1507–1515. 29124863 10.1111/ele.12854

[ece39190-bib-0051] Oksanen, J. , Blanchet, F. G. , Kindt, R. , Legendre, P. , Minchin, P. R. , O'hara, R. B. , Simpson, G. L. , Solymos, P. , Stevens, M. H. H. , Szoecs, E. , & Wagner, H. (2013). Community ecology package . R package version, 2(0).

[ece39190-bib-0052] Ollerton, J. , Winfree, R. , & Tarrant, S. (2011). How many flowering plants are pollinated by animals? Oikos, 120(3), 321–326.

[ece39190-bib-0053] Onuferko, T. M. , Skandalis, D. A. , León Cordero, R. , & Richards, M. H. (2018). Rapid initial recovery and long‐term persistence of a bee community in a former landfill. Insect Conservation and Diversity, 11(1), 88–99.

[ece39190-bib-0054] Orme, D. , Freckleton, R. , Thomas, G. , Petzoldt, T. , Fritz, S. , Isaac, N. , & Pearse, W. (2018). caper: Comparative Analyses of Phylogenetics and Evolution in R. R package version 1.0.1, https://CRAN.R‐project.org/package=caper

[ece39190-bib-0055] Pardee, G. L. , Griffin, S. R. , Stemkovski, M. , Harrison, T. , Portman, Z. M. , Kazenel, M. R. , Lynn, J. S. , Inouye, D. W. , & Irwin, R. E. (2022). Life‐history traits predict responses of wild bees to climate variation. Proceedings of the Royal Society B, 289, 20212697. 35440209 10.1098/rspb.2021.2697 PMC9019520

[ece39190-bib-0056] Potts, S. G. , Biesmeijer, J. C. , Kremen, C. , Neumann, P. , Schweiger, O. , & Kunin, W. E. (2010). Global pollinator declines: trends, impacts and drivers. Trends in ecology & evolution, 25(6), 345–353. 20188434 10.1016/j.tree.2010.01.007

[ece39190-bib-0057] Portman, Z. M. , Bruninga‐Socolar, B. , & Cariveau, D. P. (2020). The state of bee monitoring in the United States: A call to refocus away from bowl traps and towards more effective methods. Annals of the Entomological Society of America, 113(5), 337–342.

[ece39190-bib-0058] Prendergast, K. S. , Menz, M. H. , Dixon, K. W. , & Bateman, P. W. (2020). The relative performance of sampling methods for native bees: An empirical test and review of the literature. Ecosphere, 11(5), e03076.

[ece39190-bib-0059] Qian, H. , & Jin, Y. (2021). Are phylogenies resolved at the genus level appropriate for studies on phylogenetic structure of species assemblages? Plant Diversity, 43(4), 255–263. 34485767 10.1016/j.pld.2020.11.005 PMC8390917

[ece39190-bib-0060] Ramírez, S. R. , Hernández, C. , Link, A. , & López‐Uribe, M. M. (2015). Seasonal cycles, phylogenetic assembly, and functional diversity of orchid bee communities. Ecology and Evolution, 5(9), 1896–1907. 26140205 10.1002/ece3.1466 PMC4485970

[ece39190-bib-0061] Reilly, J. R. , Artz, D. R. , Biddinger, D. , Bobiwash, K. , Boyle, N. K. , Brittain, C. , Brokaw, J. , Campbell, J. W. , Daniels, J. , Elle, E. , & Ellis, J. D. (2020). Crop production in the USA is frequently limited by a lack of pollinators. Proceedings of the Royal Society B, 287(1931), 20200922. 33043867 10.1098/rspb.2020.0922 PMC7423660

[ece39190-bib-0062] Revell, L. J. (2012). Phytools: An R package for phylogenetic comparative biology (and other things). Methods Ecology and Evolution, 3, 217–223. 10.1111/j.2041-210X.2011.00169.x 23467194

[ece39190-bib-0063] Roubik, D. W. , Basset, Y. , Lopez, Y. , Bobadilla, R. , Perez, F. , & Ramírez, S. J. A. (2021). Long‐term (1979–2019) dynamics of protected orchid bees in Panama. Conservation Science and Practice, 3(12), e543.

[ece39190-bib-0064] Russo, L. , Park, M. , Gibbs, J. , & Danforth, B. (2015). The challenge of accurately documenting bee species richness in agroecosystems: Bee diversity in eastern apple orchards. Ecology and Evolution, 5(17), 3531–3540. 26380684 10.1002/ece3.1582 PMC4567859

[ece39190-bib-0065] Sluis, W. J. (2002). Patterns of species richness and composition in re‐created grassland. Restoration Ecology, 10(4), 677–684.

[ece39190-bib-0066] Stemkovski, M. , Pearse, W. D. , Griffin, S. R. , Pardee, G. L. , Gibbs, J. , Griswold, T. , Neff, J. L. , Oram, R. , Rightmyer, M. G. , Sheffield, C. S. , & Wright, K. (2020). Bee phenology is predicted by climatic variation and functional traits. Ecology Letters, 23(11), 1589–1598. 32812695 10.1111/ele.13583

[ece39190-bib-0067] Thomson, D. M. (2016). Local bumble bee decline linked to recovery of honey bees, drought effects on floral resources. Ecology Letters, 19(10), 1247–1255. 27539950 10.1111/ele.12659

[ece39190-bib-0068] Tonietto, R. K. , Ascher, J. S. , & Larkin, D. J. (2017). Bee communities along a prairie restoration chronosequence: Similar abundance and diversity, distinct composition. Ecological Applications, 27(3), 705–717. 27935661 10.1002/eap.1481

[ece39190-bib-0069] Tonietto, R. K. , & Larkin, D. J. (2018). Habitat restoration benefits wild bees: A meta‐analysis. Journal of Applied Ecology, 55(2), 582–590.

[ece39190-bib-0070] Tronstad, L. , Bell, C. , & Crawford, M. (2022). Choosing collection methods and sample sizes for monitoring bees. Agricultural and Forest Entomology. 10.1111/afe.12518

[ece39190-bib-0071] Tsirogiannis, C. , & Sandel, B. (2016). PhyloMeasures: A package for computing phylogenetic biodiversity measures and their statistical moments. Ecography, 39(7), 709–714.

[ece39190-bib-0072] Tucker, C. M. , Cadotte, M. W. , Carvalho, S. B. , Davies, T. J. , Ferrier, S. , Fritz, S. A. , Grenyer, R. , Helmus, M. R. , Jin, L. S. , Mooers, A. O. , & Pavoine, S. (2017). A guide to phylogenetic metrics for conservation, community ecology and macroecology. Biological Reviews, 92(2), 698–715. 26785932 10.1111/brv.12252 PMC5096690

[ece39190-bib-0073] Turley, N. E. , Biddinger, D. J. , Joshi, N. K. , & López‐Uribe, M. M. (2022). Six years of wild bee monitoring collections data using blue vane traps in southern Pennsylvania, USA. Dryad, 10.5061/dryad.9kd51c5mc

[ece39190-bib-0074] Webb, C. O. , Ackerly, D. D. , McPeek, M. A. , & Donoghue, M. J. (2002). Phylogenies and community ecology. Annual Review of Ecology and Systematics, 33(1), 475–505.

[ece39190-bib-0075] Williams, P. , Colla, S. , & Xie, Z. (2009). Bumblebee vulnerability: Common correlates of winners and losers across three continents. Conservation Biology, 23(4), 931–940. 19245487 10.1111/j.1523-1739.2009.01176.x

[ece39190-bib-0076] Williams, P. H. , & Osborne, J. L. (2009). Bumblebee vulnerability and conservation world‐wide. Apidologie, 40(3), 367–387.

[ece39190-bib-0077] Wilson, J. S. , Griswold, T. , & Messinger, O. J. (2008). Sampling bee communities (Hymenoptera: Apiformes) in a desert landscape: Are pan traps sufficient? Journal of the Kansas Entomological Society, 81(3), 288–300.

[ece39190-bib-0078] Winfree, R. (2010). The conservation and restoration of wild bees. Annals of the New York Academy of Sciences, 1195(1), 169–197. 20536823 10.1111/j.1749-6632.2010.05449.x

[ece39190-bib-0079] Winfree, R. , Reilly, J. R. , Bartomeus, I. , Cariveau, D. P. , Williams, N. M. , & Gibbs, J. (2018). Species turnover promotes the importance of bee diversity for crop pollination at regional scales. Science, 359(6377), 791–793. 29449491 10.1126/science.aao2117

[ece39190-bib-0080] Wood, S. N. (2017). Generalized additive models: An introduction with R (2nd ed.). Chapman and Hall/CRC.

[ece39190-bib-0081] Wood, T. J. , Gibbs, J. , Graham, K. K. , & Isaacs, R. (2019). Narrow pollen diets are associated with declining midwestern bumble bee species. Ecology, 100(6), e02697. 31012965 10.1002/ecy.2697

[ece39190-bib-0082] Woodard, S. H. , Federman, S. , James, R. R. , Danforth, B. N. , Griswold, T. L. , Inouye, D. , McFrederick, Q. S. , Morandin, L. , Paul, D. L. , Sellers, E. , Strange, J. P. , Vaughan, W. , Branstetter, M. G. , Burns, C. T. , Cane, J. , Cariveau, A. B. , Cariveau, D. P. , Childers, A. , Childers, C. , … Wehling, W. (2020). Towards a US national program for monitoring native bees. Biological Conservation, 252, 108821.

[ece39190-bib-0083] Zattara, E. E. , & Aizen, M. A. (2021). Worldwide occurrence records suggest a global decline in bee species richness. One Earth, 4(1), 114–123.

