# Supplementary material for: Six years of wild bee monitoring shows changes in biodiversity within and across years and declines in abundance
Source: Ecol Evol. 2022 Aug 12;12(8):e9190. doi: 10.1002/ece3.9190 (PMC9374588; doi:10.1002/ece3.9190)
Supplement: Supplementary file 1 — Appendix S1 [file ECE3-12-e9190-s001.docx]

# Appendix

**Table A1.** Phenological and change over time data for 40 bee species which had 30 or more individuals captured. Seasonality represents the time of year a bee is most active and is calculated as the median Julian date that each species was captured. Phenological breadth, or the length of time a species is active, is the difference between the 10th and 90th percentile of Julian day of capture. Rate of change is a measure of how much the abundance of each species changed from 2014-2019. It is measured as the model coefficient for the linear regression between year and abundance, or the slope of that relationship. Data were standardized so the coefficient represents the change in standard deviation units in abundance per year. P-value is associated with that linear regression. Values with P<0.1 are bolded.

| **Species** | **Family** | **Seasonality (median Julian day)** | **Phenological breadth (days)** | **Rate of change (model coef.)** | **P-value** |
| --- | --- | --- | --- | --- | --- |
| *Agapostemon texanus* | Halictidae | 181.9 | 109.6 | **-0.19** | **0.10** |
| *Agapostemon virescens* | Halictidae | 178.2 | 93.9 | **-0.35** | **<0.01** |
| *Andrena imitatrix* | Andrenidae | 106.4 | 19.5 | -0.16 | 0.20 |
| *Andrena miserabilis* | Andrenidae | 105.0 | 17.9 | -0.15 | 0.22 |
| *Andrena perplexa* | Andrenidae | 117.8 | 42.0 | -0.13 | 0.30 |
| *Andrena violae* | Andrenidae | 130.5 | 19.9 | 0.14 | 0.25 |
| *Anthophora bomboides* | Apidae | 169.3 | 25.4 | 0.05 | 0.67 |
| *Anthophora terminalis* | Apidae | 173.8 | 93.1 | -0.15 | 0.22 |
| *Apis mellifera* | Apidae | 178.2 | 147.1 | -0.16 | 0.17 |
| *Augochlora pura* | Halictidae | 208.6 | 97.5 | -0.10 | 0.40 |
| *Augochlorella aurata* | Halictidae | 193.8 | 73.6 | -0.11 | 0.37 |
| *Bombus bimaculatus* | Apidae | 168.9 | 45.1 | **-0.29** | **0.01** |
| *Bombus fervidus* | Apidae | 176.8 | 116.5 | **-0.31** | **0.01** |
| *Bombus griseocollis* | Apidae | 194.4 | 61.7 | 0.16 | 0.19 |
| *Bombus impatiens* | Apidae | 210.9 | 80.6 | **-0.23** | **0.05** |
| *Bombus perplexus* | Apidae | 164.2 | 25.2 | -0.15 | 0.22 |
| *Bombus vagans* | Apidae | 193.8 | 73.8 | **-0.35** | **<0.01** |
| *Calliopsis andreniformis* | Andrenidae | 200.7 | 79.4 | -0.13 | 0.30 |
| *Ceratina calcarata* | Apidae | 177.4 | 91.4 | 0.11 | 0.36 |
| *Ceratina dupla* | Apidae | 180.8 | 110.5 | -0.07 | 0.54 |
| *Ceratina mikmaqi* | Apidae | 165.9 | 100.6 | **-0.20** | **0.09** |
| *Ceratina strenua* | Apidae | 167.3 | 76.1 | 0.12 | 0.34 |
| *Eucera hamata* | Apidae | 148.4 | 31.1 | -0.12 | 0.34 |
| *Eucera pruinosa* | Apidae | 202.0 | 36.8 | -0.09 | 0.47 |
| *Halictus ligatus* | Halictidae | 209.5 | 55.2 | **-0.25** | **0.04** |
| *Hoplitis pilosifrons* | Megachilidae | 147.2 | 36.4 | 0.02 | 0.89 |
| *Lasioglossum hitchensi* | Halictidae | 183.4 | 116.5 | **-0.22** | **0.07** |
| *Lasioglossum pilosum* | Halictidae | 204.0 | 68.7 | **-0.20** | **0.09** |
| *Lasioglossum tegulare* | Halictidae | 209.5 | 98.3 | **-0.22** | **0.07** |
| *Megachile mendica* | Megachilidae | 206.2 | 71.0 | 0.10 | 0.41 |
| *Melissodes bimaculata* | Apidae | 208.7 | 35.2 | **0.44** | **<0.01** |
| *Melissodes denticulata* | Apidae | 220.7 | 36.7 | -0.02 | 0.86 |
| *Melissodes desponsa* | Apidae | 230.9 | 47.5 | **-0.36** | **<0.01** |
| *Melissodes trinodis* | Apidae | 212.8 | 43.2 | 0.14 | 0.25 |
| *Melitoma taurea* | Apidae | 212.4 | 55.1 | **-0.25** | **0.03** |
| *Osmia bucephala* | Megachilidae | 144.8 | 58.2 | -0.04 | 0.76 |
| *Osmia cornifrons* | Megachilidae | 106.3 | 21.1 | -0.10 | 0.39 |
| *Osmia pumila* | Megachilidae | 109.2 | 56.5 | 0.00 | 0.98 |
| *Ptilothrix bombiformis* | Apidae | 204.1 | 33.0 | 0.06 | 0.64 |
| *Xylocopa virginica* | Apidae | 188.9 | 88.8 | 0.16 | 0.18 |

Table A2. Bee species' natural history traits do not predict changes in abundance over time for the 40 focal species. Results of models testing if bee characteristics measured from this study (breadth, seasonality) and traits taken from the literature (body length, social vs. solitary, specialist vs. generalist diet, and below- and above-ground nesting). Phylogenetic generalized linear models account for phylogenetic relationships among species using a Brownian-motion model of trait evolution. These models were fit with the ‘pgls’ function in the ‘caper’ R package. Linear models do not account for phylogenetic non-independence among species. In the linear models social species were found to have a predicted 0.7 standard deviation units greater decline than solitary species. But this effect was not significant when accounting for phylogeny, likely because it was driven primarily by declines in Bombus species. Similarly, there was a weak relationship between phenological breadth and change over time in linear models with species with wider breadth declining more, again this effect was weaker when accounting for phylogeny.

| Phylogenetic Generalized Linear Models | | |
| --- | --- | --- |
|  |  |  |
| Variable | t | p |
| Phenological breadth | -1.33 | 0.19 |
| Seasonality | -0.69 | 0.49 |
| Body length | -1.17 | 0.25 |
| Social nesting | -0.20 | 0.84 |
| Specialist diet | -1.15 | 0.26 |
| Ground nesting | -0.15 | 0.88 |
|  |  |  |
| Linear Models |  |  |
|  |  |  |
| Variable | t | p |
| Phenological breadth | -1.83 | 0.07 |
| Seasonality | -0.22 | 0.83 |
| Body length | 1.01 | 0.32 |
| Social nesting | -2.45 | 0.02 |
| Specialist diet | 0.60 | 0.55 |
| Ground nesting | -0.49 | 0.63 |
